# Prognostic risk factors and the role of systemic inflammatory response index in predicting outcomes for non-muscle-invasive bladder cancer

**DOI:** 10.3389/fonc.2025.1646723

**Published:** 2025-09-18

**Authors:** Qi Gui, Hongwei Guo, Taiyang Liu, Xiuhua Wen, Xiang Jiao

**Affiliations:** ^1^ Department of Urology, Zhumadian Central Hospital Affiliated to Huanghuai University, Zhumadian, Henan, China; ^2^ College of Biological and Food Engineering, Huanghuai University, Zhumadian, Henan, China

**Keywords:** non-muscle-invasive bladder cancer, systemic inflammatory response index, recurrence-free survival, progression-free survival, prognostic factors

## Abstract

**Background:**

Non-muscle-invasive bladder cancer (NMIBC) presents a variable prognosis, with a significant risk of recurrence and progression. Traditional clinicopathological factors provide limited prognostic accuracy, necessitating additional biomarkers. This study aimed to evaluate the prognostic role of the systemic inflammatory response index (SIRI) and traditional risk factors in predicting outcomes in NMIBC patients.

**Methods:**

We retrospectively analyzed 158 NMIBC patients who underwent transurethral resection of bladder tumor (TURBT) between January 2021 and October 2023. Patients were stratified into recurrence/non-recurrence and progression/non-progression groups. Clinical and pathological characteristics were compared using Chi-square tests, t-tests, or Fisher’s exact tests as appropriate. Receiver operating characteristic (ROC) analysis identified the optimal SIRI cutoff, which was used for Kaplan-Meier survival analysis and Cox regression to assess independent prognostic factors for progression-free survival (PFS).

**Results:**

The optimal SIRI cutoff value for predicting progression was 0.716 (area under the curve [AUC] = 0.689, sensitivity = 0.689, specificity = 0.718). Patients with SIRI ≥ 0.716 exhibited significantly higher progression risk (P = 0.012) and poorer PFS (Log-rank P < 0.05). Multivariate Cox regression confirmed tumor count (HR = 3.273, 95% CI: 1.003–10.691, P = 0.049), primary diagnosis status (HR = 2.563, 95% CI: 1.012–7.214, P = 0.045), and high SIRI (HR = 2.979, 95% CI: 1.110–8.027, P = 0.031) as independent predictors of PFS. Recurrence analysis further revealed that high SIRI was associated with markedly increased recurrence rates in both Ta (50.0% vs. 6.2%, P < 0.001) and T1 subgroups (73.9% vs. 32.3%, P < 0.001).

**Conclusions:**

SIRI is a significant predictor of disease progression in NMIBC, although it is not associated with RFS. When combined with clinicopathological factors such as tumor stage, grade, count, and primary diagnosis status, SIRI can enhance risk stratification in NMIBC, aiding personalized management.

## 1 Introduction

Non-muscle-invasive bladder cancer (NMIBC) is one of the most common forms of bladder cancer, accounting for approximately 70-75% of all newly diagnosed bladder cancer cases. NMIBC is defined by the absence of invasion into the muscularis propria, with the tumor confined to the mucosa (stage Ta) or submucosa (stage T1) of the bladder (1–3). Despite its relatively favorable prognosis compared to muscle-invasive bladder cancer (MIBC), NMIBC remains a significant clinical challenge due to its high recurrence rates, risk of progression to muscle-invasive disease, and the substantial burden of treatment and surveillance (4, 5). Consequently, accurate prognostic assessment is crucial for risk stratification and guiding therapeutic decision-making.

Several risk factors are known to influence the prognosis of NMIBC, including tumor grade, stage, size, number, and the presence of carcinoma *in situ* (CIS). High-grade tumors, large tumors, and multifocal disease are associated with an increased likelihood of recurrence and progression (6–8). Additionally, risk factors such as age, gender, smoking history, and underlying comorbidities, including diabetes and immunosuppression, can further modify the outcome of patients with NMIBC. While these factors provide some guidance, their predictive value is often limited, and the need for more reliable biomarkers remains paramount. In recent years, the role of the systemic inflammatory response in cancer prognosis has gained considerable attention (9–11). The systemic inflammatory response index (SIRI), a novel biomarker, is an emerging prognostic tool that integrates various inflammatory markers, including the absolute neutrophil count, lymphocyte count, and monocyte count. Elevated levels of these markers have been associated with poor clinical outcomes in a variety of cancers, including bladder cancer. The SIRI has been proposed as a prognostic indicator for NMIBC, with higher values suggesting an increased risk of recurrence and progression (12, 13). Inflammatory pathways play a critical role in tumorigenesis and cancer progression, with the tumor microenvironment being heavily influenced by immune cells, cytokines, and inflammatory mediators. A chronic inflammatory state may promote tumor growth, angiogenesis, and metastasis, underscoring the potential utility of inflammatory markers as prognostic tools (14, 15).

While traditional clinical parameters have been useful in identifying high-risk NMIBC patients, the incorporation of inflammatory biomarkers such as SIRI could enhance the accuracy of risk stratification. This article aims to explore the prognostic risk factors associated with NMIBC and to assess the role of the SIRI in predicting clinical outcomes. The integration of systemic inflammatory markers into routine clinical practice may offer a promising strategy for improving the prognostication and personalized care of patients with NMIBC.

## 2 Methods

### 2.1 Study design

This retrospective cohort study was conducted at our institution to assess the prognostic risk factors and the role of the SIRI in predicting outcomes for NMIBC. The study included patients who underwent TURBT with post-operative pathological confirmation of NMIBC between January 2021 and October 2023. Inclusion criteria were: 1) Patients classified as intermediate or high risk according to the European Association of Urology (EAU) guidelines, with low-risk defined as primary, solitary TaG1 tumors ≤3 cm without carcinoma *in situ* (CIS), intermediate-risk as tumors not falling into low- or high-risk categories, and high-risk as T1 tumors, G3 (high-grade) tumors, CIS, or TaG1G2/LG tumors that were multifocal, recurrent, or >3 cm in diameter; 2) Patients who received post-operative intravesical gemcitabine chemotherapy for one year; 3) Patients with complete pre-operative laboratory assessments (complete blood count and biochemical tests) performed within one week prior to surgery; 4) Complete and detailed medical records. Exclusion criteria included incomplete clinical data, other malignancies or distant metastases, comorbidities preventing surgery, post-operative pathology showing non-NMIBC, and a history of hematologic disorders, autoimmune diseases, or recent infections. A total of 158 NMIBC patients were included. The study was conducted in accordance with the STROBE guidelines (16). Informed consent was obtained from all subjects and/or their legal guardian(s). The study was thoroughly reviewed and approved by the Ethics Committee of Zhumadian Central Hospital Affiliated to Huanghuai University and the Ethics Committee of the College of Biological and Food Engineering, Huanghuai University. It was conducted in full compliance with established guidelines and regulations, adhering to the ethical principles outlined in the Declaration of Helsinki. Data confidentiality was strictly maintained, with all personal identifiers removed prior to analysis to safeguard participant privacy.

### 2.2 Data collection

Clinical and pathological data were retrospectively collected for all enrolled patients, including demographic information such as gender and age, as well as clinical variables relevant to disease prognosis. Specifically, the following data were retrieved: TNM stage, pathological grade, tumor count, tumor diameter, whether the tumor was a first-time diagnosis or recurrent, and peripheral blood cell counts. The SIRI was calculated based on the peripheral blood cell counts, using the following formula: SIRI = (neutrophil count × monocyte count)/lymphocyte count. These parameters were used to assess the relationship between systemic inflammation and clinical outcomes in patients with NMIBC.

### 2.3 Treatment protocol

Upon admission, all patients underwent a comprehensive set of laboratory tests and diagnostic evaluations to exclude any contraindications for surgery. According to the standard protocol, patients received mTURBT (Monopolar Transurethral Resection of Bladder Tumor). Within 24 hours post-operatively, intravesical chemotherapy with epirubicin was administered. Subsequently, weekly intravesical instillations of the same drug at the same dosage were performed, alternating between epirubicin and gemcitabine throughout the treatment period, with the infusion duration extended to 1 hour per session, for a total of 8 treatments. Following the initial 8 weekly treatments, the regimen was modified to monthly instillations, continuing for a total duration of 1 year. During the treatment period, patients underwent monthly follow-up assessments, including complete blood count (CBC) and liver and kidney function tests. Cystoscopy and abdominal ultrasound were performed every 3 months during the first year, and semi-annual follow-up evaluations were conducted thereafter. This protocol was designed to monitor treatment efficacy and identify any potential complications in the management of non-muscle-invasive bladder cancer.

### 2.4 Follow-up

Follow-up was primarily conducted via telephone, supplemented by outpatient clinic visits and medical records. Recurrence-free survival (RFS) was defined as the time from the initial diagnosis, confirmed by pathological results after TURBT, to the first occurrence of tumor recurrence, progression, or death. Progression-free survival (PFS) was defined as the time from the initial diagnosis to the progression of the disease or death. Tumor progression was characterized by an increase in the T stage from Ta or T1 to T2 (muscle-invasive bladder cancer, MIBC) or higher. Both RFS and PFS were assessed based on patient survival status and clinical outcomes throughout the follow-up period.

### 2.5 Statistical analysis

Statistical analysis was conducted using SPSS software (Version 27.0). For continuous variables that followed a normal distribution, inter-group comparisons were performed using independent sample t-tests, with results presented as mean ± standard deviation. Categorical variables were expressed as frequencies and percentages, and the relationships or differences between these variables were evaluated using Chi-square (χ2) tests. When the conditions for χ2 testing were not met, Fisher’s exact test was employed. To assess whether the SIRI values differed across groups, receiver operating characteristic (ROC) curves were constructed. The optimal cutoff value for SIRI was determined by calculating the maximum Youden index from the ROC curve. Based on this cutoff, patients were classified into distinct groups. Survival analysis was conducted using the Kaplan-Meier method, and inter-group comparisons were performed with the Log-rank test. Age was categorized into two groups (<60 years and ≥60 years) for inclusion in the Cox regression analysis. Both univariate and multivariate Cox proportional hazards models were applied to identify independent prognostic factors for NMIBC, with the assumption that the proportional hazards assumption was met for all variables. All hypotheses were two-tailed, and statistical significance was defined as a p-value of less than 0.05.

## 3 Results

### 3.1 Clinical characteristics by recurrence and progression status

A total of 158 patients with non-muscle-invasive bladder cancer (NMIBC) were included in this study. Patients were categorized into recurrence and non-recurrence groups, as well as progression and non-progression groups, based on post-operative outcomes. A comparative analysis of the clinical and pathological characteristics between these groups is presented in **Table 1**.

**Table 1:** Comparison of baseline clinical and pathological characteristics between recurrence and non-recurrence groups, and progression and non-progression groups.

Clinical and pathological characteristics	Recurrence group (n=56)	Non-recurrence group (n=102)	P	Progression group (n=38)	Non-progression group (n=120)	P
Gender (Male/Female)	49 (87.5%)/7 (12.5%)	73 (71.6%)/29 (28.4%)	0.038	35 (92.1%)/3 (7.9%)	91 (75.8%)/29 (24.2%)	0.030
Tumor Diameter (<3/≥3)	34 (60.7%)/22 (39.3%)	68 (66.7%)/34 (33.3%)	0.427	19 (50.0%)/19 (50.0%)	83 (69.2%)/37 (30.8%)	0.076
Number of Tumors (single/2-7/≥8)	20 (35.7%)/26 (46.4%)/10 (17.9%)	65 (63.7%)/25 (24.5%)/12 (11.8%)	0.012	10 (26.3%)/18 (47.4%)/10 (26.3%)	75 (62.5%)/27 (22.5%)/18 (15.0%)	0.002
T Stage (Ta/T1)	12 (21.4%)/44 (78.6%)	69 (67.6%)/33 (32.4%)	<0.001	6 (15.8%)/32 (84.2%)	75 (62.5%)/45 (37.5%)	<0.001
Grade (G1/G2/G3)	6 (10.7%)/11 (19.6%)/39 (69.6%)	27 (26.5%)/54 (52.9%)/21 (20.6%)	<0.001	4 (10.5%)/3 (7.9%)/31 (81.6%)	29 (24.2%)/58 (48.3%)/33 (27.5%)	<0.001
Primary Diagnosis (Yes/No)	44 (78.6%)/12 (21.4%)	96 (94.1%)/6 (5.9%)	<0.001	22 (57.9%)/16 (42.1%)	107 (89.2%)/13 (10.8%)	<0.001

G, Grade;

T, Tumor stage.

The mean age of patients in the recurrence group was 57.16 ± 11.68 years, while that of the non-recurrence group was significantly higher at 62.78 ± 8.28 years (P < 0.001). The recurrence group had neutrophil, lymphocyte, monocyte, and platelet counts of 3.71 ± 1.29, 2.09 ± 0.66, 0.41 ± 0.12, and 218.68 ± 62.89 × 10^9/L, respectively. The SIRI value for this group was 0.81 ± 0.39. In comparison, the non-recurrence group had counts of 3.66 ± 1.39 for neutrophils, 2.01 ± 0.58 for lymphocytes, 0.36 ± 0.11 for monocytes, and 239.36 ± 52.68 × 10^9/L for platelets, resulting in a mean SIRI of 0.70 ± 0.46. Significant differences were observed between the recurrence and non-recurrence groups in terms of age (P < 0.001), gender (P = 0.022), tumor stage (P < 0.001), tumor grade (P < 0.001), primary diagnosis status (P < 0.001), and monocyte count (P = 0.029), indicating that these factors may contribute to the likelihood of recurrence in NMIBC patients.

Patients in the progression group had a mean age of 65.09 ± 10.98 years, which was significantly higher than that of the non-progression group, which had a mean age of 58.16 ± 9.16 years (P < 0.001). Neutrophil, lymphocyte, monocyte, and platelet counts in the progression group were 4.01 ± 1.51, 2.08 ± 0.58, 0.46 ± 0.09, and 218.39 ± 61.89 × 10^9/L, respectively, with a mean SIRI value of 0.91 ± 0.51. For the non-progression group, these values were 3.59 ± 1.36 for neutrophils, 2.11 ± 0.68 for lymphocytes, 0.36 ± 0.11 for monocytes, and 229.58 ± 53.28 × 10^9/L for platelets, resulting in a SIRI of 0.71 ± 0.39. Significant differences between the progression and non-progression groups were observed for age (P < 0.001), gender (P = 0.030), tumor count (P = 0.002), tumor stage (P < 0.001), tumor grade (P < 0.001), primary diagnosis status (P = 0.002), monocyte count (P < 0.001), and SIRI (P = 0.012). These findings suggest that higher age, advanced tumor stage, higher tumor grade, and elevated SIRI are associated with disease progression.

### 3.2 Relationship between SIRI and recurrence-free survival

The SIRI was analyzed to assess its association with RFS in patients with NMIBC. The mean SIRI value in the recurrence group was 0.81 ± 0.39, while the mean SIRI in the non-recurrence group was 0.70 ± 0.46. Statistical analysis revealed no significant difference in SIRI values between these two groups (P = 0.132), indicating that SIRI was not significantly associated with RFS in this cohort.

### 3.3 Optimal SIRI cutoff value for predicting disease progression

**Figure 1:**
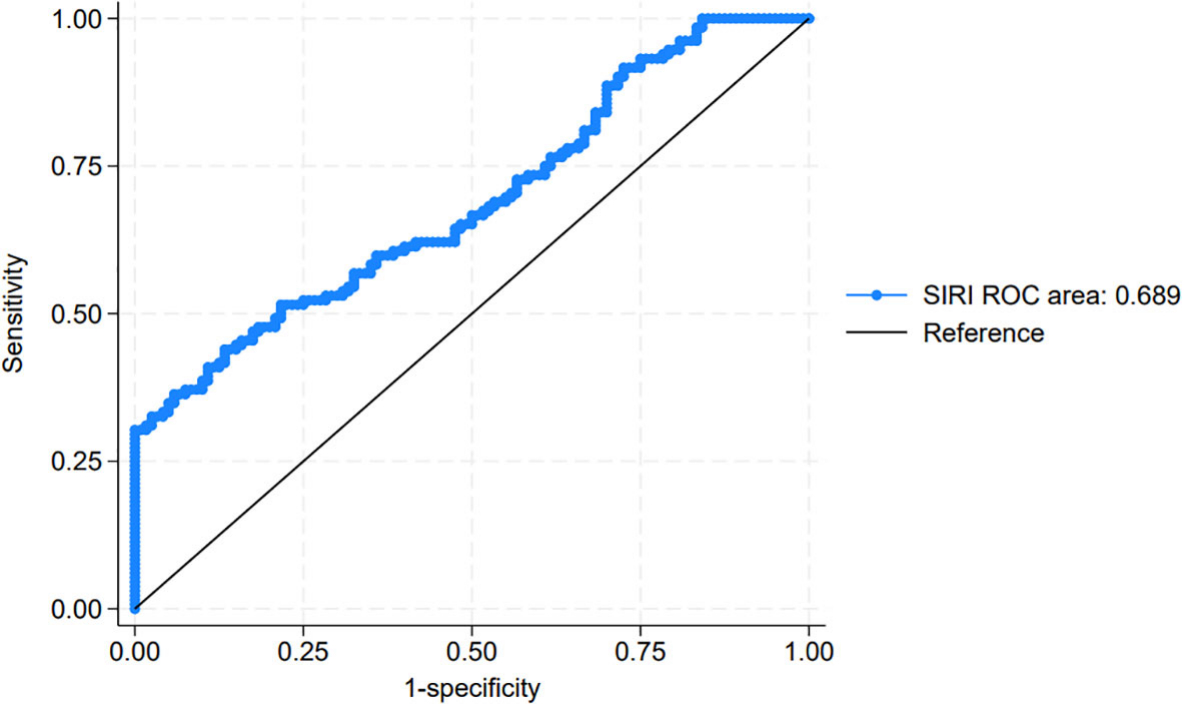
Receiver operating characteristic (ROC) curve for systemic inflammatory response index (SIRI) in predicting post-operative progression in non-muscle-invasive bladder cancer (NMIBC).

To evaluate the prognostic significance of the SIRI in predicting disease progression in NMIBC, we compared SIRI values between the progression and non-progression groups. The mean SIRI in the progression group was significantly higher at 0.91 ± 0.51 compared to 0.71 ± 0.39 in the non-progression group (P = 0.012), suggesting that elevated SIRI may be associated with an increased risk of progression. Using ROC curve analysis, we identified the optimal SIRI threshold for predicting post-operative disease progression (**Figure 1**). The area under the curve (AUC) was 0.689, indicating moderate discriminative ability. The optimal cutoff point for SIRI, defined by the maximum Youden index (0.358), was determined to be 0.716. At this threshold, the sensitivity was 0.689 and the specificity was 0.718, providing a balanced measure for distinguishing between patients with and without progression. Based on this cutoff, patients were stratified into two groups: the low SIRI group (SIRI < 0.716, n = 96) and the high SIRI group (SIRI ≥ 0.716, n = 62).

### 3.4 Association between SIRI and progression-free survival

**Figure 2:**
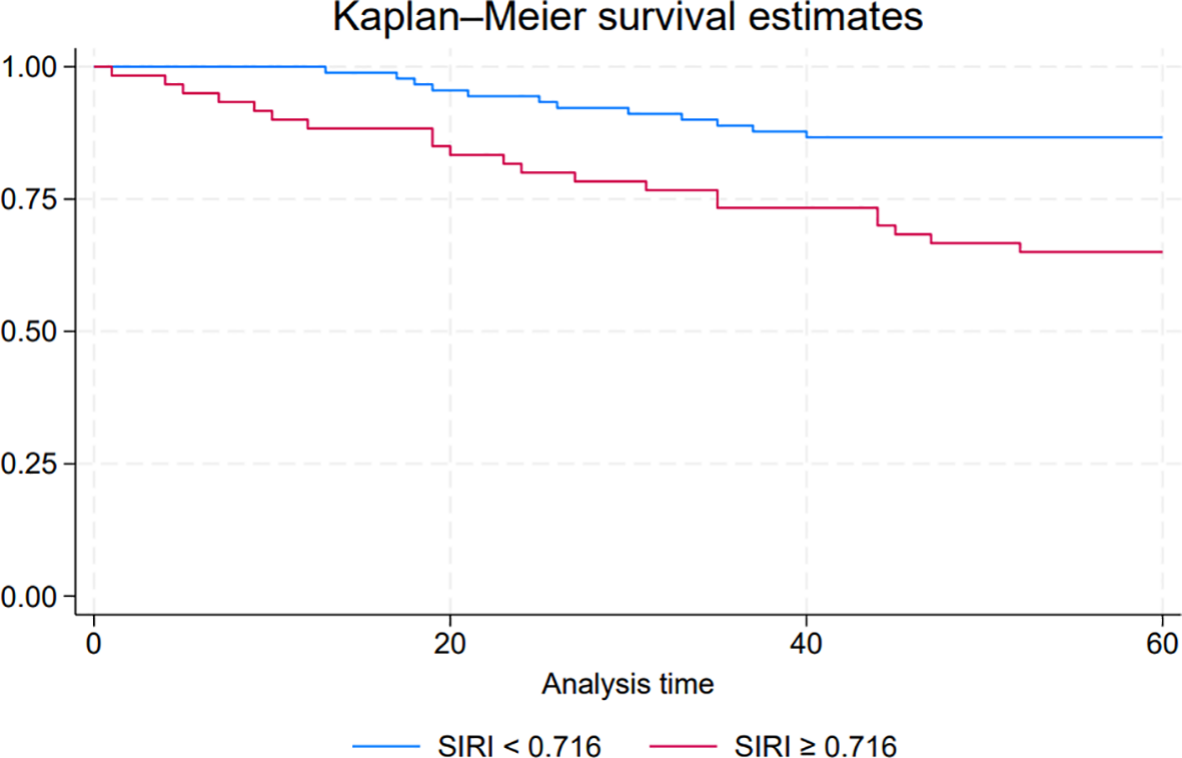
Kaplan-Meier survival curves comparing progression-free survival (PFS) between low systemic inflammatory response index (SIRI) and high SIRI groups in NMIBC patients.

To explore the impact of the SIRI on PFS in NMIBC patients, we conducted a Kaplan-Meier survival analysis comparing PFS between the low SIRI group (SIRI < 0.716) and the high SIRI group (SIRI ≥ 0.716). The survival curves for PFS, shown in **Figure 2**, illustrate a statistically significant difference between these two groups (P < 0.05). Patients in the low SIRI group demonstrated significantly better PFS outcomes compared to those in the high SIRI group. These findings suggest that a lower SIRI is associated with a reduced risk of disease progression, supporting the potential prognostic value of SIRI as an indicator for PFS in NMIBC.

### 3.5 Cox regression analysis of factors influencing PFS

A Cox proportional hazards regression analysis was performed to identify factors influencing PFS in patients with NMIBC following TURBT. In the univariate Cox regression model, several variables were identified as significant risk factors for reduced PFS, including age, tumor count, tumor stage, tumor grade, primary diagnosis status, and a SIRI value ≥0.716 (**Table 2**). These variables were subsequently included in a multivariate Cox regression analysis to determine independent prognostic factors. The results indicated that tumor count, primary diagnosis status, and a SIRI value ≥0.716 remained as independent predictors of PFS in NMIBC patients (**Table 3**).

**Table 2:** Univariate analysis of factors influencing PFS in NMIBC patients.

Clinical pathological characteristics	HR	95% CI	P
Age (<60 vs. ≥60 years)	2.639	1.058 – 6.191	0.046
Gender (Female vs. Male)	6.788	0.891 – 49.864	0.061
Tumor Diameter (<3 cm vs. ≥3 cm)	1.915	0.801 – 4.552	0.123
Tumor Count (2–7 vs. Single)	2.083	0.726 – 6.119	0.151
Tumor Count (≥8 vs. Single)	5.512	1.839 – 16.724	0.003
T Stage (Ta vs. T1)	6.043	1.986 – 17.855	0.002
Grade (G2 vs. G1)	0.285	0.045 – 1.647	0.180
Grade (G3 vs. G1)	3.655	1.045 – 12.095	0.041
Primary Diagnosis (No vs. Yes)	4.170	1.711 – 10.068	0.002
SIRI (Low vs. High)	3.238	1.288 – 8.001	0.011

HR, Hazard Ratio.

CI, Confidence Interval.

PFS, Progression-Free Survival.

SIRI, Systemic Inflammatory Response Index.

G, Grade.

T, Tumor stage.

**Table 3:** Multivariate Cox regression analysis of factors influencing PFS in NMIBC patients.

Clinical pathological characteristics	HR	95% CI	P
Age (<60 vs. ≥60 years)	1.216	0.394 – 3.801	0.713
Tumor Count (2–7 vs. Single)	1.452	0.485 – 4.341	0.503
Tumor Count (≥8 vs. Single)	3.273	1.003 – 10.691	0.049
T Stage (Ta vs. T1)	1.765	0.213 – 13.101	0.597
Grade (G2 vs. G1)	0.231	0.036 – 1.381	0.142
Grade (G3 vs. G1)	1.444	0.193 – 10.568	0.729
Primary Diagnosis (No vs. Yes)	2.563	1.012 – 7.214	0.045
SIRI (Low vs. High)	2.979	1.110 – 8.027	0.031

HR, Hazard Ratio.

CI, Confidence Interval.

PFS, Progression-Free Survival.

SIRI, Systemic Inflammatory Response Index.

G, Grade.

T, Tumor stage.

### 3.6 Recurrence rates stratified by tumor stage according to SIRI

Analysis of recurrence rates stratified by T stage and SIRI levels demonstrated that, within the Ta subgroup, patients with SIRI ≥ 0.716 had a markedly higher recurrence rate compared to those with SIRI < 0.716 (50.0% [8/16] vs. 6.2% [4/65]; χ² = 19.56, P < 0.001) (**Table 4**). Similarly, in the T1 subgroup, the recurrence rate was significantly higher in patients with SIRI ≥ 0.716 than in those with SIRI < 0.716 (73.9% [34/46] vs. 32.3% [10/31]; χ² = 13.12, P < 0.001). These findings indicate that elevated SIRI is associated with an increased recurrence risk across both early (Ta) and more advanced (T1) stages of NMIBC.

**Table 4:** Recurrence rates stratified by T stage and tumor grade according to SIRI levels in NMIBC patients.

Variable	Subgroup	SIRI < 0.716 Recurrence, n/N (%)	SIRI ≥ 0.716 Recurrence, n/N (%)	χ²	P-value
T stage	Ta	4/65 (6.2%)	8/16 (50.0%)	19.56	<0.001
	T1	10/31 (32.3%)	34/46 (73.9%)	13.12	<0.001

T, Tumor stage; SIRI, systemic inflammatory response index.

## 4 Discussion

SIRI integrates elevated neutrophils and monocytes together with lymphopenia and thus captures a systemic, pro-tumor inflammatory milieu (17, 18). Neutrophils can promote invasion via release of proteases and neutrophil extracellular traps and by sustaining IL-6/STAT3 and NF-κB signaling; circulating monocytes differentiate toward tumor-associated macrophages that support angiogenesis, extracellular-matrix remodeling, and immune evasion; conversely, lymphopenia reflects impaired anti-tumor surveillance (19, 20). These processes are biologically more relevant to tumor progression (i.e., acquisition of muscle invasion and aggressive growth) than to local recurrence, which in NMIBC is often driven by intraluminal re-implantation, residual microscopic disease after TURBT, and field cancerization. This mechanism-based distinction may explain why SIRI was independently associated with PFS but not with RFS in our cohort. If validated, a high SIRI could be used to flag patients who may benefit from intensified management aimed at reducing progression risk: closer cystoscopic surveillance; early repeat TURBT when indicated; preference for more effective intravesical immunotherapy in eligible patients; and comprehensive optimization of modifiable inflammatory burdens (e.g., treating urinary infections, smoking cessation, metabolic control). Whether targeted anti-inflammatory or immunomodulatory interventions (e.g., strategies that modulate myeloid-cell recruitment or restore lymphocyte competence) can improve outcomes in high-SIRI patients remains to be tested prospectively; our data should be viewed as hypothesis-generating.

In our study, SIRI was not significantly associated with RFS, in contrast to prior findings on inflammatory markers such as NLR and PLR. This divergence may stem from distinct underlying mechanisms: recurrence in NMIBC is likely driven by local factors—such as field cancerization, intraluminal seeding, and adherence to intravesical therapy—rather than systemic inflammation. Therefore, SIRI may be insufficient to capture the biological processes responsible for recurrence (21, 22). Conversely, elevated SIRI was significantly associated with disease progression. Patients in the progression group exhibited higher mean SIRI values, and ROC analysis identified 0.716 as the optimal cutoff, demonstrating moderate discriminatory ability. Kaplan-Meier analysis further confirmed that patients with high SIRI had significantly shorter progression-free survival. These findings suggest that a heightened systemic inflammatory response may contribute to tumor progression through mechanisms such as immune evasion, angiogenesis, and tumor proliferation (23, 24). Importantly, multivariate Cox regression identified SIRI ≥ 0.716, tumor count, and primary diagnosis status as independent predictors of progression. The prognostic value of SIRI likely reflects an immunosuppressive state characterized by elevated neutrophils and monocytes relative to lymphocytes, disrupting antitumor immune surveillance. As a readily available and cost-effective biomarker, SIRI may aid in identifying NMIBC patients at high risk for progression who could benefit from intensified monitoring or early therapeutic escalation.

An elevated SIRI, identified as an independent predictor of progression, may serve as a complementary biomarker to refine NMIBC management. High-SIRI patients exhibited substantially higher recurrence rates in both Ta (50.0% vs. 6.2%) and T1 (73.9% vs. 32.3%) stages, indicating that systemic inflammation exerts a prognostic impact on recurrence independent of pathological stage. This likely reflects tumor–host interactions that facilitate tumor cell survival, reimplantation, and progression. Clinically, high SIRI may justify intensified surveillance protocols (e.g., shortened cystoscopic intervals, early re-TURBT) and consideration of more aggressive intravesical regimens, including maintenance immunotherapy. In intermediate-risk disease, SIRI could inform earlier treatment escalation under shared decision-making, while in very-high-risk phenotypes, it may lower the threshold for discussing early radical cystectomy. Given that SIRI reflects a pro-inflammatory and immunosuppressive state, optimization of modifiable inflammatory drivers—such as infection control, management of metabolic comorbidities, and smoking cessation—remains relevant (25, 26). Although the benefit of targeted anti-inflammatory or immunomodulatory interventions is unproven, our findings are hypothesis-generating and warrant prospective validation, ideally integrating SIRI with established clinicopathological risk factors.

Our findings are supported by Bizzarri et al., who also demonstrated that elevated preoperative SIRI was significantly associated with recurrence and progression in NMIBC patients. While their study used a separate cohort and confirmed the prognostic value of SIRI using ROC and Cox models, external validation using public datasets such as TCGA remains lacking across studies, including ours. Therefore, prospective validation and integration with large-scale, publicly available genomic and clinical databases remain critical for future research (27). Several recent studies have highlighted the prognostic relevance of SIRI in bladder cancer. Ye et al. (1) demonstrated that high pretreatment SIRI was associated with poorer BCG response and shorter recurrence-free and progression-free survival in NMIBC patients, supporting our finding that elevated SIRI independently predicts progression. Yilmaz et al. (28)further validated SIRI as an independent predictor of recurrence and overall survival in MIBC patients undergoing radical cystectomy, suggesting that the prognostic utility of SIRI extends across disease stages. Compared with these studies, our work reinforces the prognostic value of SIRI in a TURBT-treated NMIBC cohort, even among patients not receiving BCG, and highlights its role as a systemic biomarker complementing conventional clinicopathological factors.

Functional relevance and limitations of SIRI. In our cohort, SIRI showed a moderate ability to predict progression (AUC = 0.689; sensitivity = 0.689; specificity = 0.718) but was not associated with RFS. Biologically, SIRI integrates neutrophilia and monocytosis together with lymphopenia, capturing a systemic pro-tumor inflammatory and immunosuppressive state. Myeloid-driven pathways (e.g., proteases/NETs, IL-6/STAT3 and NF-κB signaling, macrophage-mediated angiogenesis and matrix remodeling) are plausibly linked to invasive growth and immune evasion—mechanisms more relevant to progression—whereas recurrence in NMIBC is frequently driven by local factors such as residual disease, re-implantation, and field cancerization after TURBT. Despite statistical significance for PFS, SIRI alone has important limitations as a biomarker. It is non-specific and may vary with acute/chronic infections, smoking, metabolic and inflammatory comorbidities, or medications (e.g., corticosteroids, hematopoietic stimulators); although we excluded recent infections and hematologic/autoimmune disorders, residual confounding cannot be ruled out. SIRI also reflects a single preoperative time point; dynamic changes around surgery or during intravesical therapy were not captured. The optimal cutoff was derived internally and requires external validation; assay and laboratory variability may affect transportability. Finally, the AUC indicates only moderate discrimination, so SIRI is unlikely to be clinically actionable in isolation (29). Integration with composite models. Given these constraints, SIRI is better conceptualized as one component of a multivariable risk tool. Our dataset includes complete blood counts, enabling derivation of other inflammatory ratios (e.g., NLR, PLR, SII). Future work should evaluate whether combining SIRI with clinicopathological factors (stage, grade, tumor count, and primary vs. recurrent diagnosis—variables that were significant in our Cox models) improves discrimination and clinical utility, using DeLong’s test for AUC comparison, calibration plots, decision-curve analysis, and reclassification metrics (NRI/IDI). Such a composite score or nomogram could enhance individualized risk stratification beyond traditional factors alone.

Our findings have several clinical implications. First, SIRI may serve as a practical biomarker for stratifying NMIBC patients according to their risk of progression, enabling a more tailored approach to patient management. Patients with elevated SIRI values may require more frequent cystoscopic surveillance, consideration of adjuvant intravesical therapies, or enrollment in clinical trials for novel interventions targeting inflammation. Additionally, integrating SIRI into risk assessment models could improve the prognostic accuracy of existing NMIBC guidelines, potentially enhancing clinical decision-making. However, it is important to acknowledge the limitations of this study, including its retrospective design and single-institution setting, which may limit generalizability. Further large-scale, prospective studies are needed to validate the role of SIRI in NMIBC and to elucidate the specific inflammatory mechanisms contributing to disease progression. In summary, while SIRI was not predictive of recurrence in NMIBC, it demonstrated potential as an independent prognostic marker for progression, offering a promising avenue for improving risk stratification and personalized care in this patient population.

## 5 Conclusions

This study demonstrates that while SIRI is not significantly associated with RFS in NMIBC, an elevated SIRI (≥0.716) is a significant predictor of disease progression and poorer PFS. SIRI, along with clinicopathological factors such as tumor stage, grade, tumor count, and primary diagnosis status, may serve as a valuable biomarker for identifying high-risk NMIBC patients, supporting more personalized management strategies.

## Data Availability

The raw data supporting the conclusions of this article will be made available by the authors, without undue reservation.
